# Common variable immunodeficiency and nodular lynphoid hyperplasia: case report

**DOI:** 10.1186/1939-4551-8-S1-A216

**Published:** 2015-04-08

**Authors:** Erica Sbrissa, Estela Risso, Maria Elisa Andrade, João Ferreira De Mello, Cintia Bassani, Dayane Brandini, Tatiana Mercuri De Campos

**Affiliations:** 1 Hospital Servidor Público Estadual De São Paulo, Brazil

## Background

Nodular lymphoid hyperplasia (NLH) of intestine is an extremely rare lymphoproliferative disorder of uncertain etiology. Usually, polyps benign lymphoid tissue are present in the intestinal mucosa. It may present as an asymptomatic or manifest with gastrointestinal symptoms such as abdominal pain, chronic diarrhea, occult bleeding or intestinal obstruction. NLH found in association with common variable immunodeficiency (CVI) constitutes an increased risk for malignant transformation.

## Methods

Report a patient with common variable immunodeficiency and diffuse nodular lymphoid hyperplasia of the small and large intestine.

## Results

RG, 32, male, valued at outpatient consultation in Gastroenterology, Hospital do Servidor Público Estadual de São Paulo in January 2014 due to heartburn and reflux. As personal history, had undergone total colectomy in 2009 for multiple polyps at colonoscopy and diagnosis of familial adenomatous polyposis. Pathology revealed reactive follicular lymphoid hyperplasia. Evolved with loose and frequent stools without blood or mucus. Upper Digestive Endoscopy in 2009: enanthematic pangastritis and light bulboduodenite. A sigmoidoscopy conducted in 2010 showed ileal polyps and ileo-rectal anastomosis with inflammatory pseudo-polyps. Was admitted for further investigation with diagnoses of Inflammatory Bowel Disease and Lymphoproliferative Disease. The abdominal CT revealed lymph node enlargement in several chains and chest CT without evidence of lymph node enlargement. Colonoscopy showed sessile polyps in the ileum and colon mucosa with micronodular pattern. Metronidazole received due to *Giardia lamblia* in feces. The dosage of immunoglobulins IgG showed 279mg/dl, and IgA and IgM decreased, the diagnosis of CVI being taken. Received intravenous gammaglobulin 400mg/kg. Duodenal biopsy revealed nonspecific chronic inflammation with immunohistochemistry and search for *Giardia lamblia* negative. The revision blade colectomy reiterated the diagnosis of follicular lymphoid hyperplasia. The patient follow-up in outpatients receiving monthly intravenous infusion of gammaglobulin.

## Conclusions

We have reported a case of a patient with CVI that presented NLH. Due to increased risk for intestinal lymphoma, should be kept under constant surveillance by tumor tracking.

## Consent

Written informed consent was obtained from the patient for publication of this abstract and any accompanying images. A copy of the written consent is available for review by the Editor of this journal.

